# Global datasets of geospatial-AI-resolved energy consumers including climate-driven energy demands, geographical and socioeconomic realities for a transition reset

**DOI:** 10.1038/s41597-024-04277-x

**Published:** 2024-12-19

**Authors:** Diego Moya, Dennis Copara, Sara Giarola, Adam Hawkes

**Affiliations:** 1 Energy Traceability Technology, Technology Strategy and Planning Department, Saudi Aramco, Dhahran, 34481 Saudi Arabia; 2 Department of Chemical Engineering, Imperial College London, South Kensington, London, SW7 2BX UK; 3 Institute for Applied Sustainability Research, IIASUR, Quito, 170806 Ecuador; 4 School of Management, Milan, 20156 Italy; 5 RFF-CMCC EIEE, Milan, 20144 Italy

**Keywords:** Energy and behaviour, Energy economics

## Abstract

Traditional models deliberately simplify millions of consumers into a single, homogeneous, representative agent with perfect market knowledge and rational expectations, limiting their capacity to capture real-world complexities. To address this limitation in mainstream models, this article provides global datasets to parametrise energy consumers within climate-energy-economy models considering climate-driven energy demand, socioeconomic and demographic factors. The datasets emerge from applying geospatial artificial intelligence, machine learning and big data analytics on a range of geospatial parameters at 1 km^2^ resolution. Twenty distinctive energy consumers are defined using three heterogeneous geospatial features, eight diverse and two evolving parameters. This parametrisation of consumers strengthens the applicability of climate-energy-economy models to guide effective, equitable and just climate policy design. This comprehensive analysis of complex interactions between climate, socioeconomic and demographic factors supports more realistic decision-making for a sustainable transition reset. This research emphasises the geospatial distribution of energy consumers to enhance technoeconomic assessment, understanding consumer dynamics for consumer-led resource allocation and informed policy implementation. These datasets can be used in climate-energy-economy models to parametrise consumers beyond traditional approaches.

## Background & Summary

Representing energy consumers in the assessment of complex transition of the climate-energy-economy systems is not an easy task. Geospatial artificial intelligence (geoAI) combining with agent-based modelling (ABM) foundations has emerged as a potential approach to characterise energy consumers as autonomous, evolving (in space and time), and adaptive decision-making entities with prescribed bounded behavioural rules, neither perfect nor imperfect (but actual behaviour)^1^. These consumers can interact with the environment and other consumers to generate emergent system-level patterns (e.g., of energy consumption)^2^, under exogenous constraints, as found in Moya, *et al*.^3^ and Crooks, *et al*.^4^. The combination of geoAI and ABM is rooted in complex adaptive systems (CAS) theory, which views complex systems as the result of interactions among lower-level components^5^. GeoAI arises as a key application of artificial intelligence (AI) combined with geospatial data and big data analytics to accelerate real-world understanding and characterisation of energy consumers and their environmental impacts of their decision-making process^3^.

The key principles of ABM to represent the human dimension in complex climate-energy-economy systems include (i) heterogeneity of agents, (ii) diversity of agents and (iii) agent evolution, derived from CAS in complex systems science^4^. These principles can guide the understanding of agent interactions in climate-energy-economy using ABM under bounded rationality theory^6,7^. (i) Heterogeneity captures the agent boundaries for their aggregate behaviour. By defining agent heterogeneity, physical shaping structure can be characterised. These structures include agent restrictions such as income, energy demand, and energy consumption that can influenced agent decision-making^8^. These restrictions can be modelled to define the boundaries of agent interaction, determining space and time borders. Once defined agent heterogeneity, (ii) agent diversity parameters can be calculated within the previously defined boundaries of agents’ interactions^9^. Examples of agent diversity include the Human Development Index (HDI), population (POP) count, POP densities, and Gross Domestic Product per capita (GDPpc), among others. These parameters can be applied to specific groups of heterogeneous agents previously characterised. By linking heterogeneity and diversity, models establish the boundaries of agent interactions based on heterogenous characteristics and parameterise a range of diverse metrics within those boundaries. For those diverse agents interacting within specific boundaries, estimating (iii) the agent evolution represents the changing profile of parameters within the defined boundaries allows for the representation of agent evolution over space and time^10^. This is the most challenging aspect to calculate. Data availability for this characteristic is often limited, with few countries providing high spatiotemporal resolution datasets^11^. Historical data is typically available at the city or country level, not at the individual agent level. Estimating future profiles of agent characteristics remains difficult. However, some initiatives such as the Shared Socioeconomic Pathways (SSPs)^12^ can provide disaggregated data on POP and GDP across different income levels and age ranges under various scenarios, offering insights into future agent profiles.

Spatiotemporal datasets along big data analytics and AI tools (e.g., machine learning, ML) are continuously expanding, posing challenges for researchers while offering significant importance and benefits to stakeholders^13^. Geospatial big data analytics is being employed by urban planners, local authorities, enterprises, researchers, and energy policymakers to gain insights from these vast amounts of geospatial data. One area of focus is utilizing geospatial big data analytics to enhance sustainable energy transitions^14^. It can provide valuable information to stakeholders regarding the development of sustainable energy strategies, including energy policies, energy developments, and energy research. However, several information challenges still hinder progress. Barriers such as data availability, scalability, integration, inconsistency, geocoding, and privacy need to be addressed to fully unleash the potential of geospatial big data analytics in climate-energy-economy models^15^. Although progress is being made in overcoming these challenges, understanding the spatiotemporal variations of various factors influencing energy consumption in the energy sector, in general, and particularly in residential sector modelling remains an unexplored area in the literature.

geoAI offers promising techniques for integrating and comparing relevant features in the planning of sustainable energy transitions of the residential sector^16^. Geospatial data mining and geoAI play a crucial role in extracting hidden knowledge from large, gridded datasets, providing valuable insights for decision-makers^17^. ML algorithms, statistical methods, and AI methods are utilized in geospatial big data analytics to address complex energy problems^18^. Unsupervised ML techniques are commonly employed in energy analysis, allowing for the classification of spatial data points into specific groups based on similar properties. The goal of clustering is to group similar data points together based on their features. For instance, the DBSCAN (Density-Based Spatial Clustering of Applications with Noise) algorithm is used to disaggregate electrical load profiles into space heating (SH) and water heating (WH)^19^. Hierarchical cluster analysis is applied to develop energy transition policies for sustainable urban areas^20^. The K-means algorithm is utilized to assess global gridded energy demand density^21^. These methods help uncover hidden patterns, detect anomalies, and simplify data for further analysis, making them valuable in various fields of energy studies such as energy demand segmentation, and gridded data analysis. However, existing studies primarily focus on analysing spatial big data without incorporating its use in climate-energy-economy modelling. In the energy sector, clustering methods have been extensively employed, particularly in recognizing patterns of electricity consumption. In Voulis, *et al*.^22^, a comprehensive review of clustering techniques used for assessing spatiotemporal electricity demand profiles at urban scales is provided.

**Fig. 1:**
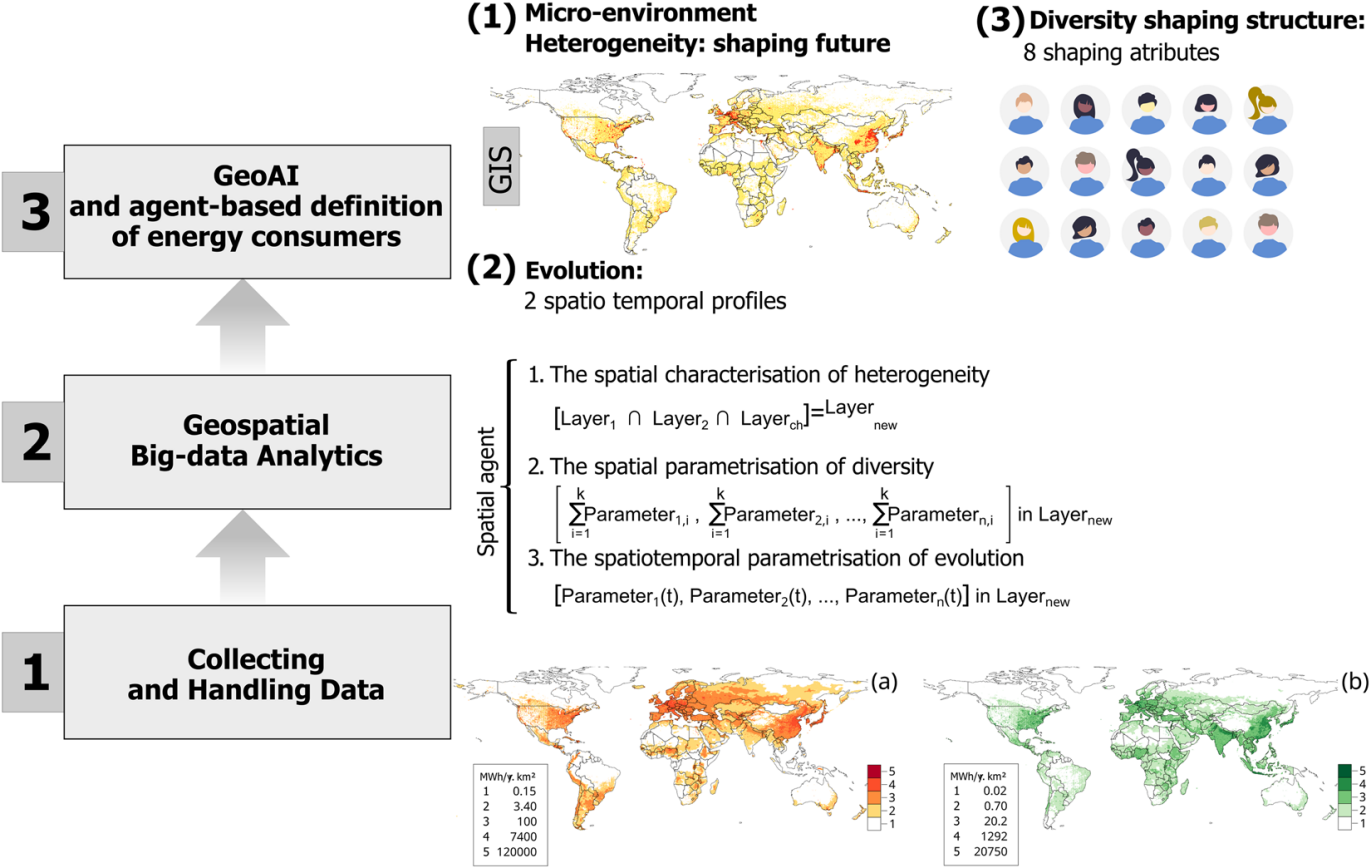
Study design to obtain global datasets of geospatial-AI-resolved energy consumers or agents including energy, geographical and socioeconomic parameters. Examples of data collection for Step 1 are: (**a**) Space heating, SH, (**b**) Space cooling, SC. In total, ten global gridded datasets were used in this study, as explained in Moya, *et al*.^3^.

This research has been designed to systematically and methodologically characterising and parametrising energy consumers, also called agents, to serve as energy inputs in climate-energy-economic models. To achieve this, this research has applied geoAI and ABM foundations and tools. A recent study in Moya, *et al*.^3^ serves as starting point to end with the datasets presented here. This data descriptor article aims to share a dataset collection to characterised and parametrised energy consumers at the global level, aggregating data into 28 regions worldwide (See Supplementary Information). Characteristics of energy consumers are captured at a 1 km^2^ resolution, including geographical, climate and socioeconomic conditions across borders. Eleven parameters are used to present the dataset collection to geospatially parametrise agent heterogeneity (three parameters), agent diversity (eight parameters), and agent evolution (two parameters). These datasets can be easily introduced in the complex transition assessment of climate-energy-economic systems. Figure 1 illustrates a schematic overview of the study design for this research.

This article aims to geospatially define and parametrise energy economic agents, consumers, as an alternative option to the hyper-rational representation of an optimum, homogenous, representative agent as described by Shaikh^8^, which is typical of traditional energy systems models that are based on neoclassical economic foundations, the mainstream^23,24^. The representation overcomes the limitations of considering consumers as rational and homogeneous representative agents that optimise utility, previously discussed by Simon^7^, Bardazzi and Bosello^25^, Karjalainen, *et al*.^26^, Shaikh^8^ and others^27^. To overcome the forementioned limitations of traditional models, the consumer definition of this article is based on three groups of attributes: (1) the complex nature of agent heterogeneity in terms of three geospatial characteristics, (2) the complex characteristics of agent diversity in terms of eight geospatial parameters, and (3) the complex agent evolution in terms of geo-spatiotemporal profiles of two socioeconomic macro-drivers. After describing the gap in this section, the definition of the agents, their attributes, their geographical characterisation, their parametrisation, the temporal socioeconomic evolution of agents are further discussed in the novel methodology presented here. A technical validation of the method is then presented in the last part after the data records sections, followed by the conclusion and discussion. The geospatial agent definition presented in this article captures the complexities of representing agents of a socio-technical and economic system, considering non-technical and non-economic factors, within a climate-energy-economy model of the residential energy sector.

## Methods

Overall, these datasets presented here have been computed from the geospatial reclassified datasets presented in Moya, *et al*.^3^ and Moya, *et al*.^28^. Energy consumers heterogeneity can be defined using two or three geospatial attributes. Then, consumer diversity and evolution parameters are estimated accordingly in each heterogenous zone defined previously. Detailed consumer evolution can be found in Moya, *et al*.^28^. The methodology used in this research is an extension of the MUSE-RASA (ModUlar energy system Simulation Environment, MUSE, ResidentiAl Spatially-resolved and temporal-explicit Agents, RASA) framework methodology presented in Moya, *et al*.^3^. geoAI-based clustering analysis is used for geospatial data mining to discover hidden patterns and re-classify data into groups called clusters. These geospatial clusters are the primary characteristics of consumers to define the borders where these consumers interact and share similar characteristics. K-means method was explored and applied. As K-means algorithm holds significant potential for energy demand studies^29^, it was extensively used in this study. However, determining the number of clusters (*k*) in the K-means approach poses a challenge. The Elbow Method (EM) was applied to evaluate the within-cluster variance as a function of k and determine the optimal number of clusters (*ONC*), as explained in Zhang, *et al*.^30^. This research uses spatiotemporal data to calculate the ONC by computing the sum of squared errors (*SSE*) between cluster centres and their members. The plot of *k* against *SSE* allows for visually identifying the *ONC* based on the “elbow” of the curve^21^. This method supports the selection of number of clusters for further characterisation of consumers.

Overall, 10 geospatial (also known as gridded) datasets are required. Gridded energy demand datasets [(i) SH, (ii) WH, (iii) SC, and (iv) TE for heating and cooling, TE] at 1 km^2^ hourly-seasonal resolution, were collected from Sachs, *et al*.^21^. Gridded datasets for (v) heating demand density, HD, and (vi) cooling demand density, CD, were collected from Moya, *et al*.^31^. Global socioeconomic, development, and demographic gridded datasets [(vii) GDP, (viii) GDPpc, (ix) HDI, and (x) POP count] at 1 km^2^ resolution were collected from Kummu, *et al*.^32^ and calibrated using geospatial datasets from Center for International Earth Science Information Network - Columbia University (CIESIN)^33^. The only limitation has been identified in terms of geospatial resolution for HDI. HDI was collected at the city or province level depending on data availability.

In the next three steps, the general description of the geospatial-AI-resolved agent definition framework is provided. The heterogeneity, diversity, and evolution of agents are defined using geo-AI, geospatial big-data analytics and ABM foundations. Abstraction from the real world to characterise and parametrise energy consumers is provided. These datasets can be applied at the micro- and macro-environments of any climate-energy-economic model. After applying these energy consumer definitions, model outcomes and policy development can capture the three characteristics of energy consumers presented here: heterogeneity, diversity, and evolution.

### Geospatial-AI characterization of energy consumer heterogeneity (3 attributes)

**Fig. 2:**
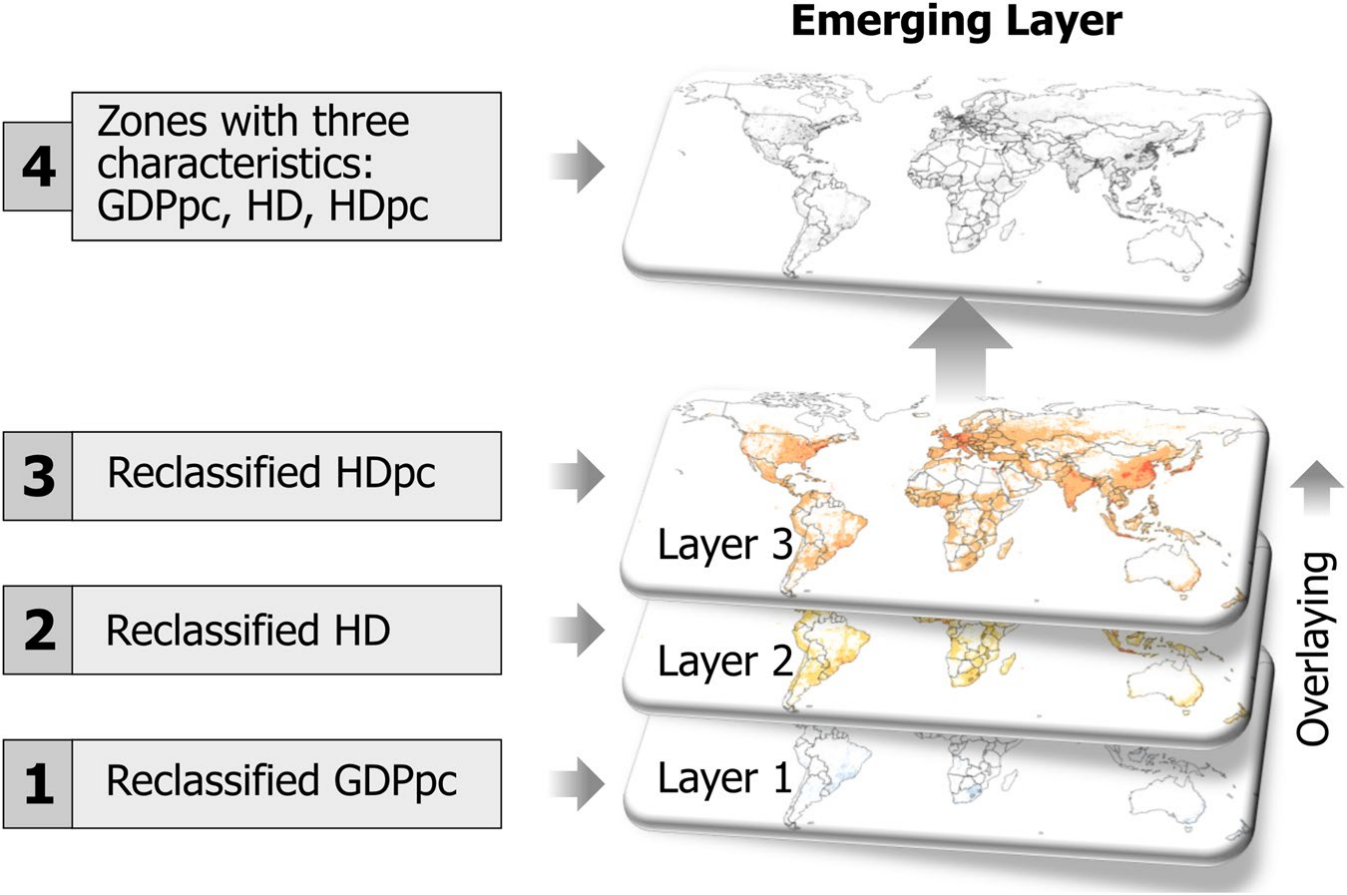
Gridded GDPpc, HD and HDpc are initially reclassified applying geoAI-ML techniques. Then, GDPpc, HD and HDpc are overlayed to spatially characterised agent heterogeneity with three geospatial attributes.

For the characterisation of energy consumer heterogeneity, geoAI methods are applied to three geospatial attributes: GDPpc, heat demand per capita (HDpc), heat density (HD). HDpc, is calculated using SH demand and POP. Equation 1 and Fig. 2 summarise the methods for the energy consumer heterogeneity characterisation. Equation 1 defines the spatial agent (SpA) using three spatial characteristics. Gridded 1 km^2^ resolution GDPpc, HD and HDpc are initially reclassified using ML and geospatial big data techniques from the geoAI domain. Then, reclassified GDPpc, HD and HDpc are geospatially overlayed to geospatially characterised agent heterogeneity with three geospatial attributes. The overlaying process produces an emergent layer that captures the shaping structures of energy consumer heterogeneity. From the overlaying emerges a new layer of zones used to estimate the datasets presented in this study. Table 1 presents the bounds for GDPpc, HDpc and HD.

1 $${Characteristic}\,1=\left[\left({{SpA}}_{{{GDP}}_{{PC}}}\cap {{SpA}}_{{{HD}}_{{PC}}}\right)\cap {{SpA}}_{{HD}}\right]$$

Where:

- ${{SpA}}_{{{GDP}}_{{PC}}}$ represents the spatial agents defined by GDPpc.
- ${{SpA}}_{{{HD}}_{{PC}}}$ represents the spatial agents defined by HDpc.
- ${{SpA}}_{{HD}}$ represents the spatial agents defined by HD.

**Table 1:** GDPpc-based energy consumer classes.

	GDPpc [USD/cap*yr]	
	Lower bound	Upper bound
GDPpc1	min	500
GDPpc2	500	3785
GDPpc3	3785	18125
GDPpc4	18125	41667
GDPpc5	41667	75901
GDPpc6	75901	max
	HDpc [MWh/cap*yr]	
	**Lower bound**	**Upper bound**
HDpc1	min	0.9
HDpc2	0.9	3.2
HDpc3	3.2	5.3
HDpc4	5.3	max
	**HD [MWh/km**^2^*yr]	
	**Lower bound**	**Upper bound**
HD1	min	1790
HD2	1790	12080
HD3	12080	36930
HD4	36930	max

GDPpc1 and GDPpc6 classes have been defined based on the literature, and the four remaining classes are the result of a geoAI-ML K-means clustering approach, explained in Moya, *et al*.^3^. Estimated annual HDpc extreme classes (HDpc1 and HDpc4) are based on literature^3^. Heat density (HD) classes based on previously clustered heat density data are explained and published in Moya, *et al*.^3^.

### Geospatial-AI parametrisation of energy consumer diversity (8 attributes)

For the parametrisation of energy consumer diversity, geoAI methods are applied to eight geospatial attributes: GDPpc, TE, SH, WH, SC, POP count, POP density and HDI. Once, the geospatial layer of heterogeneity zones is defined using GDPpc, HDpc, and HD, the total values of eight attributes are systematically extracted from each new zone, k. Equation 2 illustrates the aggregated parameters to define the diversity characteristic of energy consumers. The aggregate GDP is calculated using $\mathop{\sum }\limits_{i=1}^{k}{{GDP}}_{i}$ and represents the total aggregate GDP in each 3-attribute characterised zone i, from zone 1 to zone-k. The aggregate TE is calculated using $\mathop{\sum }\limits_{i=1}^{k}{{TE}}_{i}$ and represents the total end-use energy service demand in each 3-attribute characterised zone. The aggregate SH is calculated using $\mathop{\sum }\limits_{i=1}^{k}{{SH}}_{i}$ and represents the total consumer SH demand in each 3-attribute characterised zone. The aggregate WH is calculated using $\mathop{\sum }\limits_{i=1}^{k}{{WH}}_{i}$ and represents the total consumer WH demand in each 3-attribute characterised zone. The aggregate SC is calculated using $\mathop{\sum }\limits_{i=1}^{k}{{SC}}_{i}$ and represents the total consumer cooling demand in each 3-attribute characterised zone. The aggregate POP count is calculated using $\mathop{\sum }\limits_{i=1}^{k}{{POP}}_{i}$ and represents the total number of energy consumers in each 3-attribute characterised zone. The POP share is calculated using $\frac{\mathop{\sum }\limits_{i=1}^{k}{{POP}}_{i}}{{TPOP}}$ and represents the share of energy consumers in each 3-attribute characterised zone. The median HDI share is calculated using ${\overline{{HDI}}}_{i}$ and represents the Median HDI in each 3-attribute characterised zone.

2 $${Characteristic}\,2=\left[\mathop{\sum }\limits_{i=1}^{k}{{GDP}}_{{pc},i},\mathop{\sum }\limits_{i=1}^{k}{{TE}}_{i},\mathop{\sum }\limits_{i=1}^{k}{{SH}}_{i},\mathop{\sum }\limits_{i=1}^{k}{{WH}}_{i},\mathop{\sum }\limits_{i=1}^{k}{{SC}}_{i},\mathop{\sum }\limits_{i=1}^{k}{{POP}}_{i},\,\frac{\mathop{\sum }\limits_{i=1}^{k}{{POP}}_{i}}{{TPOP}}{,\overline{{HDI}}}_{i}\right]$$

Where:

- ${{GDP}}_{{pc},i}$ represents the total GDPpc in each zone *i* to *k*.
- ${{TE}}_{i}$ represents the TE in each zone *i* to *k*.
- ${{SH}}_{i}$ represents the total SH in each zone *i* to *k*.
- ${{WH}}_{i}$ represents the total WH in each zone *i* to *k*.
- ${{SC}}_{i}$ represents the total SC in each zone *i* to *k*.
- ${{POP}}_{i}$ represents the total POP count in each zone *i* to *k*.
- ${TPOP}$ represents the total POP in each region.
- ${\overline{{HDI}}}_{i}$ represents the median HDI in each zone *i* to *k*.

### Geospatial-AI parametrisation of energy consumer evolution (2 attributes)

For the parametrisation of energy consumer evolution, geoAI methods are applied to two geospatial attributes: GDP and POP count. Once, the geospatial layer of heterogeneity zones is defined using GDPpc, HDpc, and HD, the evolution in space and time of GDP, and POP count is systematically estimated for each new zone, from *i* to *k*. The geo-spatiotemporal parametrisation of agent evolution (3) is captured according to the assumptions in the SSP2 narrative for region-based macroeconomic drivers, GDP^34^ and population^35^ profiles. Each agent follows a region path, depending on their specific location, independently of the geospatial characteristics. Thus, an agent with the same GDPpc, HD, and HDpc characteristics would evolve differently over time, depending on the actual region patterns where the agent is located. Equation 3 illustrates the definition of the evolution characteristic of energy consumers.

3 $${Characteristic}\,3=\left[{GDP}\left(t\right),{POP}\left(t\right)\right]$$

Where:

- ${GDP}\left(t\right)$ represents the GDP profile in time for each region or country.
- ${POP}\left(t\right)$ represents the POP profile in time for each region or country.

## Data Records

Table 1 summarises the five datasets presented in this study. Overall, the format used to report these datasets is csv. The variables presented in each dataset are described in Table 2.

**Table 2:** geo-AI-ML-resolved datasets provided in this article and their names in the repository [https://figshare.com/s/f774ff42ac4fb2b5a730]^38^.

Dataset	Name in repository	Format	Coverage	Variables
Clustered energy consumers’ parameters	01_Global_geoAI_energy_consumer_parameters_clusters	.csv	28 regions, globally 96 agents	GDP, GDPpc, ET, SH, SC, WH, POP, HDI
Sub clustered energy consumers’ parameters	02_Global_geoAI_energy_consumer_parameters_sub-clusters	.csv	28 regions, globally 20 agents	GDP, GDPpc, ET, SH, SC, WH, POP, HDI
Sub clustering validation	03_Global_geoAI_validation_subclustering_ave_sil_width	.csv	28 regions, globally	Percentage of well grouped dataAverage Silhouette width
Sub clustering errors	04_Global_geoAI_validation_subclustering_errors_28regions	.csv	28 regions, globally	Error for GDP, GDPpc, ET, SH, WH, POP, HDI
Sub clustering WSS	05_Global_geoAI_validation_wss_num_clusters	.csv	28 regions, globally	ONC, WSS

### Clustered energy consumers’ parameters

**Fig. 3:**
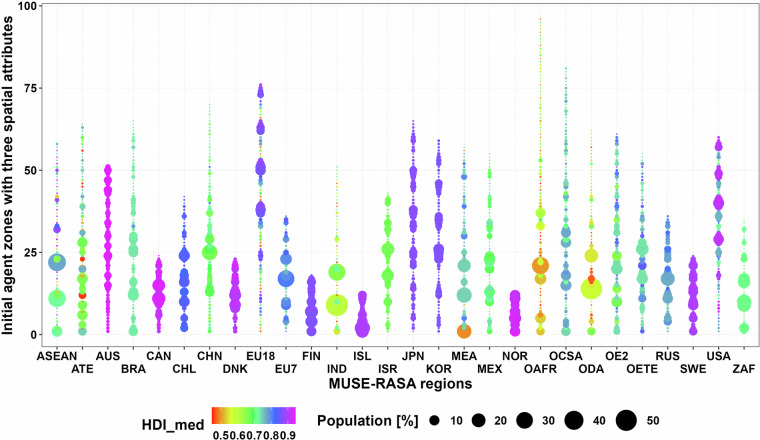
Initial heat consuming agents for the 28 MUSE-RASA model regions, considering three geospatially resolved attributes. The circle area represents the population share of each agent in each region. The HDI is reflected in a colour scale.

This dataset [01_Global_geoAI_energy_consumer_parameters_clusters.csv] provides eight parameters to define up to 96 consumers globally when considering three heterogenous spatial features. Energy consumers (A: agents) are defined using lower and upper bounds of GDPpc, HD and HDpc, as shown in Table 1. These consumers are geographically distributed and identified for each region, globally. This dataset also present key values of HDI for each energy consumer as shown in Fig. 3.

### Sub clustered energy consumers’ parameters

**Fig. 4:**
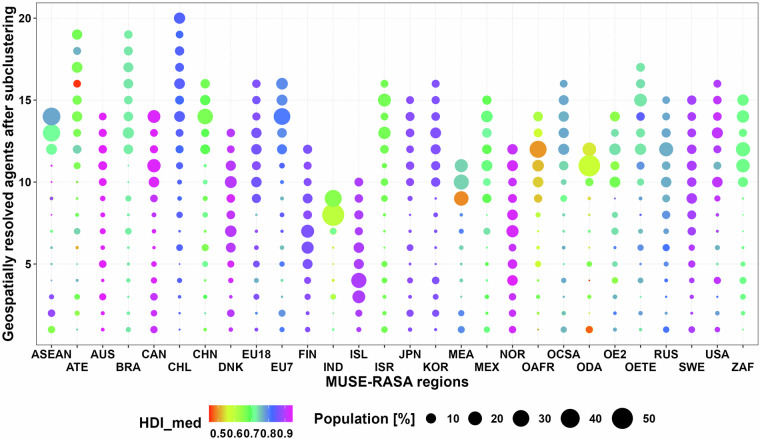
Final heat consuming agents for the 28 MUSE-RASA model regions after subclustering in terms of HDI and total population.

This dataset [02_Global_geoAI_energy_consumer_parameters_sub-clusters.csv] also provides eight parameters to define up to 20 consumers globally when considering a subclustering to the previous 96 global energy consumer agents. Figure 4 identifies these energy consumers in terms of HDI and total population after sub clustering to distinguish the difference with previous dataset.

**Fig. 5:**
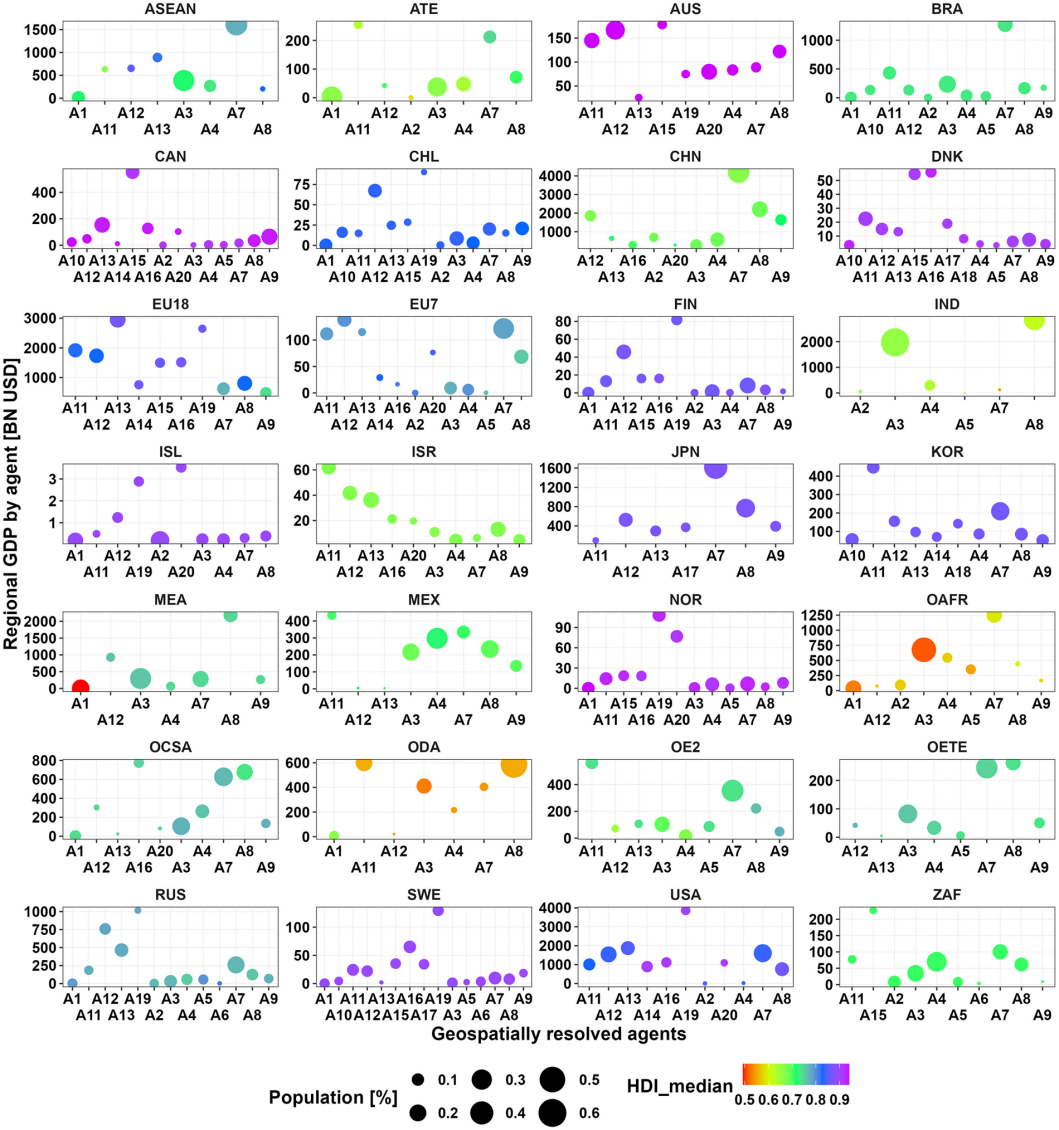
Region-based disaggregation of Gross Domestic Product, Human Development Index and population share for geospatial energy consumers parametrisation or agent (A).

Figure 5 provides the 20 energy consumers in terms of GDP, HDI and total POP after sub clustering in each region of the world. These parameters are also included in this dataset.

### Sub clustering validation

**Fig. 6:**
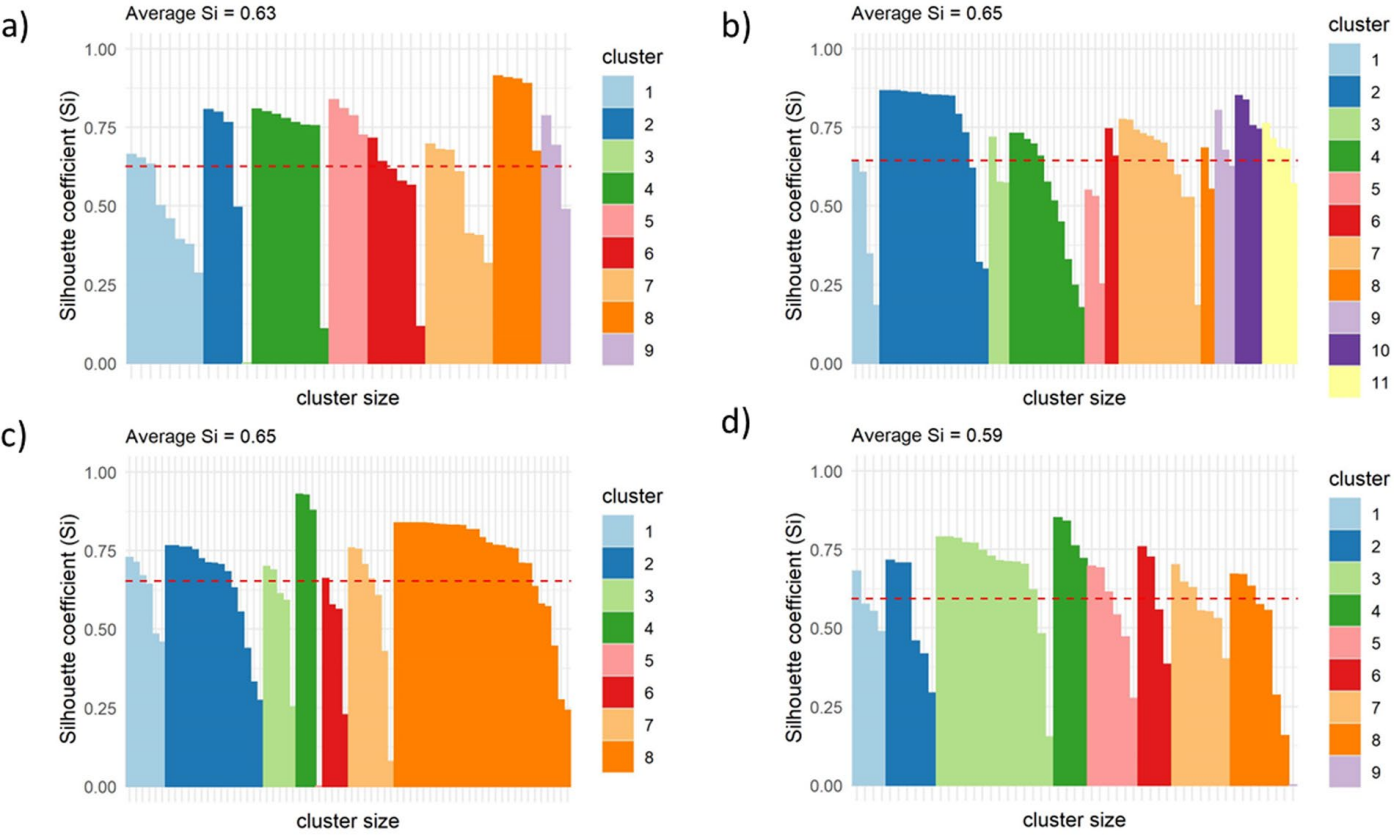
Silhouette coefficient (silhouette width) to evaluate the goodness of spatial agent subclustering in selected regions: (**a**) Australia, (**b**) China, (**c**) EU18, and (**d**) USA.

This dataset [03_Global_geoAI_validation_subclustering_ave_sil_width.csv] provides the subclustering goodness of the final spatial agents or energy consumers. The Silhouette coefficient (Silhouette width) is reported for each consumer in each region, as can be seen in Fig. 6 for four selected regions. This dataset presents three key variables: (i) percentage of well grouped data, (ii) the average Silhouette width, and (iii) the total number of clusters under analysis. This dataset presents the three variables for the 28 regions of study.

### Sub clustering errors

This dataset [04_Global_geoAI_validation_subclustering_errors_28regions.csv] provides the error between the values of the energy consumer parametrisation and the aggregated parameter at the regional level. Errors at the national level where estimating aggregating the values at the cluster level. The error was calculated for GDP, GDPpc, TE, SH, WH, POP.

### Sub clustering WSS

**Fig. 7:**
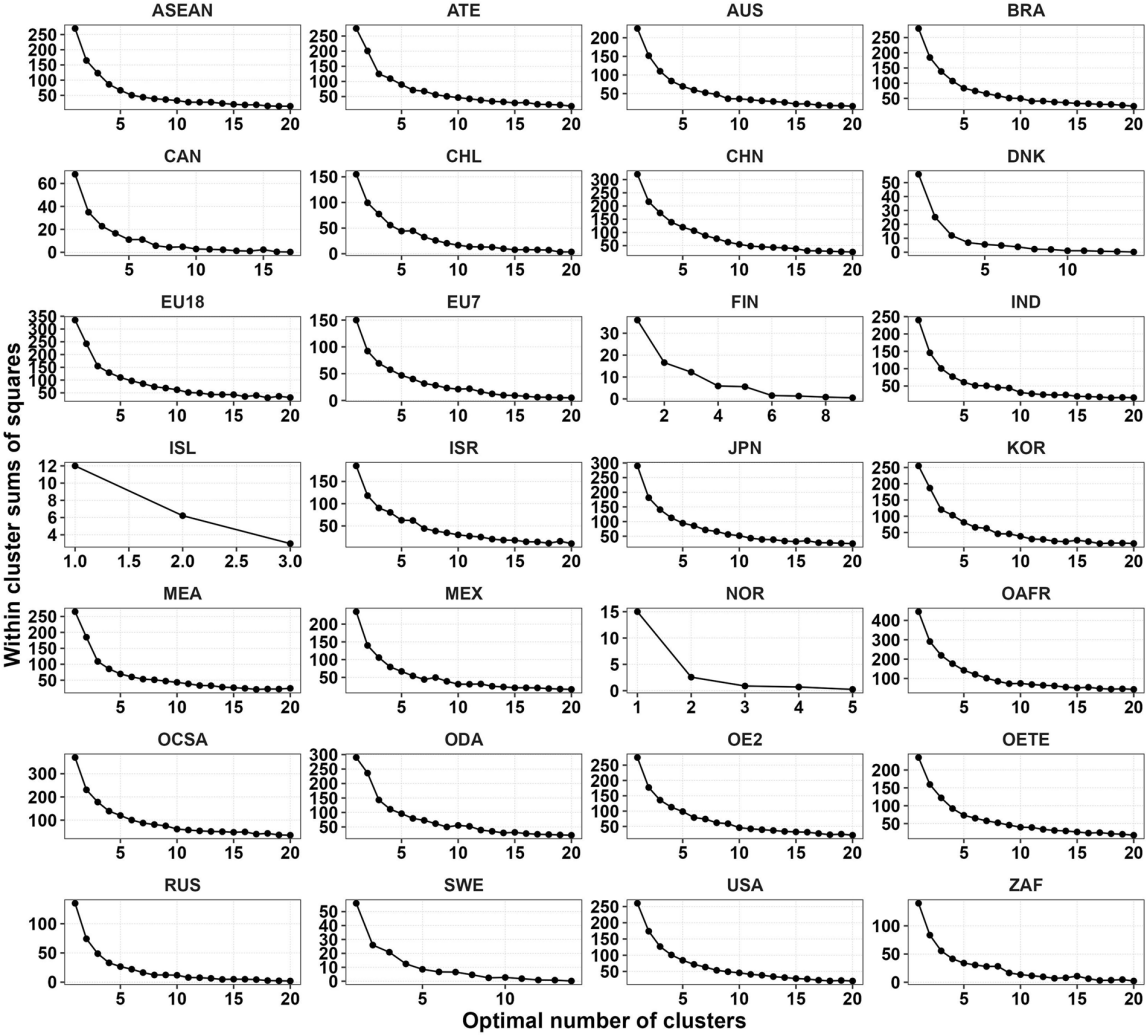
EM for the subclustering approach of heating-based agents. EM to define the optimal number of agents in each region. This number of agents is used as an input in the K-means algorithm to group agents. The evolution of SSE values with increasing the number of clusters, k creates an elbow that suggests a cluster solution.

This dataset [05_Global_geoAI_validation_wss_num_clusters.csv] provides the values of the Within-Cluster-Sum of Squared Errors (WSS) for each clusters k in each region, as observed in Fig. 7. With WSS, it is possible to apply the EM in order to determine k that later serves to define the number of energy consumers with a range of characteristics.

## Technical Validation

The validation process of the geoAI-resolved energy consumers at various scales, from national to global, poses a significant challenge and requires the integration of Geographic Information Systems (GIS), big data analytics, geoAI methods and ABM foundations. In this research, a meticulous cross-validation of the global geospatial definition of energy consumers was conducted at three stages of geoAI-based calculations. These validation processes were implemented for the agent subclustering approach (compactness and goodness) and the agent parametrisation approach (error).

The subclustering approach reduced the number of energy consumers globally from 96 to 20, showcasing the effectiveness of the EM in determining the ONC. The results indicated a high percentage of well-grouped data, with a BSS/TSS ratio greater than 0.975 for each region, demonstrating that over 97% of the initial 96 agents were effectively grouped into 20 agents. This meticulous subclustering is crucial as it captures specific zones of GDPpc, HD, and HDpc, characterising them with a range of parameters for subsequent stages of the research.

Figure 6 is also part of the validation, which illustrates the goodness of the subclustering using the Silhouette coefficient, confirming the ONC for selected regions. The absence of negative Silhouette width and average Silhouette coefficients ranging from 0.59 to 0.69 reinforce the effectiveness of the agent subclustering, ensuring well-grouped and optimally clustered energy consumers. These Silhouette coefficient values can be considered acceptable for gridded energy data, particularly when this data is based on geospatial population distribution across nations. This is because energy data inherently contains noise and outliers, reflecting the diverse and uneven distribution of populations and energy consumption patterns. Variations in economic development, infrastructure, and energy access contribute to these irregularities, making perfect clustering challenging. As a result, the presence of noise and outliers can skew the results, leading to moderately high Silhouette scores in this range. Additionally, varying cluster densities, where densely populated areas have smaller intra-cluster distances compared to sparsely populated regions, further impact the Silhouette scores. Given the complexities and heterogeneity of global energy data, a Silhouette coefficient between 0.59 and 0.69 indicates that the clustering algorithm has reasonably captured the underlying patterns despite the inherent variability in the data. Thus, these values are acceptable and reflect the real-world intricacies of gridded energy data. The thickness analysis of the Silhouette coefficient for each agent in each region provides an additional criterion for evaluating the goodness of the subclustering.

In the third validation process, the dataset 5 outlines the agent parametrisation validation, presenting the error between agent parametrisation values and aggregated parameters at the regional level. The results indicate a global measure of error that is largely satisfactory, with some regions achieving less than 1% error. Overall, this validation underscores the robustness of the agent parametrisation approach, as it minimises errors in most agents and regions. These validation processes collectively affirm the reliability and accuracy of the geoAI approach, establishing a strong foundation for their application and relevance in understanding the global dynamics of places and people (consumers) in the context of energy transition and sustainability research.

The data quality of the original sources used in this study are confirmed to follow the standards of robustness and credibility, as it derives from well-established, peer-reviewed publications. For energy-related data, our team initially published the data and methods in the Applied Energy Journal^21^, which has undergone rigorous peer review and validation, ensuring the reliability and accuracy of the information. For socioeconomic data, this article utilised data from a Scientific Data article within the Nature Portfolio, known for its high standards in data descriptor articles^32^. These sources provide a strong foundation for the study, ensuring the data’s integrity and the validity of the subsequent analyses and conclusions drawn.

This article introduces novel contributions to the energy field by defining geospatial energy consumers agents on a global scale through the integration of three groups of attributes: heterogeneity, diversity, and evolution. It identifies twenty distinct agents worldwide by overlaying three geospatial characteristics and quantifying eight geospatial parameters for each agent within every region, using ten geospatial datasets in total. The evolution of these agents is analysed using two socioeconomic macroeconomic drivers from the SSP2 narrative. This comprehensive framework aids in understanding and analysing the geospatial dynamics of consumers globally, integrating ABM and geoAI-based approaches to represent the complexities of agent heterogeneity and diversity, adding realism to models.

The research incorporates views from Simon, Shaikh, and Crooks to represent consumers through heterogeneity, diversity, and evolution through agent shaping structures. Socio-economic shaping structures, such as income levels, energy consumption, and propensity to consume, are used to define agent boundaries and interactions. The study estimates agent diversity characteristics and tracks their evolution over time for GDP and population. The datasets show that defining energy economic agents with geospatial attributes using big data analytics provides a realistic definition of consumers for a more realistic global energy sector’s decarbonisation. The identification of 20 agent along with their populated zones, and their parameters, such as GDP, GDPpc, HDI and energy demand, enables a systematic approach to modelling complex socio-technical-economic systems, aiding stakeholders in evaluating long-term energy planning and transitions.

Validation of spatial consumers linking space and time is crucial due to the complexity of potentially millions of consumers. This study addresses the challenge through comprehensive cross-validation at three stages of GIS-based calculations, including gridded heating and cooling demands validation for two countries at 1 km^2^ resolution, agent subclustering validation, and agent parametrisation error estimation. The subclustering approach significantly reduces heating demand agents globally from 96 to 20, ensuring high compactness. The evaluation of subclustering goodness using the Silhouette coefficient indicates optimal clustering for regions such as the USA, China, EU18, and Japan. Although the geospatial definition of economic agents demanding cooling is provided, further research is needed to evaluate the technology and fuel transition of household heating and cooling worldwide. This research enhances the reliability and robustness of geospatial agent-based models, providing valuable insights for stakeholders evaluating long-term energy planning and transitions.

Using 20 energy consumers globally to assess the energy transition presents several potential weaknesses. Firstly, although most of the models only use one hyper-rational representative energy consumer, the approach presented here may still oversimplify the complex socio-economic and geographical variations inherent in energy consumption patterns. This is potentially leading to a generalised representation that may not capture local nuances effectively, mostly because of the subclustering that reduced consumers from 96 to 20. The initial 96 consumers may be adequately better represented in terms of regional variability in energy demand, influenced by factors such as climate, culture, economic development, and infrastructure; this may not be adequately reflected by a small number of 20 agents. This limitation could undermine the accuracy and precision of estimations of other transition metrics across different regions such as consumption, emissions and costs. Moreover, decision-making based on aggregated data from these agents might overlook localised needs even though this research makes sure to define zones with similar geospatial characteristics. Aggregated analysis leads to hindering the development of tailored energy policies and planning strategies responsive to potential misrepresented specific regional conditions. Additionally, validating results derived from the 20 agents across diverse regions globally poses challenges due to differences in data availability, quality, and local contexts, which could affect the reliability and robustness of findings. Lastly, the static nature of the agent-based approach may struggle to capture dynamic changes in energy demand patterns over time, limiting its effectiveness in predicting long-term trends and informing adaptive energy planning strategies.

Regarding future research, while the initial study did not explicitly present the tuning process for the model or identify the best hyperparameters, this remains an important area for future research. The energy-related data was calibrated based on the IEA energy balances, as explicitly considered in the original data sources. For validation purposes, the datasets for two countries were independently calibrated and tuned. Hyperparameters can indeed be regionally dependent due to varying socio-economic and geospatial characteristics across different areas. Each of the 28 regions analysed may benefit from customised hyperparameter settings to enhance model performance and accuracy. For further modelling and utilisation of these datasets, defining hyperparameters can simplify the learning process and provide better control over the model’s behaviour. Future work will focus on systematically tuning the model for each region, employing techniques such as grid search or Bayesian optimization, to identify the optimal hyperparameters that best capture the unique attributes and dynamics of each region.

## Usage Notes

This dataset is shared for non-commercial use only. The dataset owner, Diego Moya, reserves the right to use this data for future commercial purposes. Any commercial use, redistribution, or derivative works require prior written permission from the owner. This ensures transparency and protects the dataset’s intended purpose. This dataset is made available under the CC BY-NC-SA 4.0 license (Attribution-NonCommercial-ShareAlike). Non-commercial use is permitted with proper attribution. Commercial use, redistribution, or derivative works require prior written permission from the dataset owner.

The datasets presented in this article are of potential use in planning the decarbonisation of the energy sector when considering energy consumers characteristics such as heterogeneity, diversity, and evolution. The parametrisation of energy consumers using geoAI and ABM, along with the integration of 10 geospatial datasets described here, can significantly enhance the accuracy and comprehensiveness of climate, energy, and economic models. There are at least ten potential uses of the datasets presented here:

1. *Fine-grained geospatial resolution*: The use of 1 km² hourly seasonal resolution gridded energy demand datasets for SH, WH, SC, and total energy demand (TE) allows for a fine-grained understanding of energy consumption patterns. This level of detail is crucial for capturing localized variations in energy demand, which is essential for accurate modelling. The datasets presented here capture the spatiotemporal variability of the demand in the heterogeneity and diversity definitions.
2. *Spatial distribution of energy demand:* Gridded datasets for heating demand density (HD) and cooling demand density (CD) provide insights into the spatial distribution of energy demand at a granular level. This information is valuable for identifying areas with high energy demand, allowing policymakers and stakeholders to target interventions more effectively. HD and CD are potential metrics to evaluate the techno-economic feasibility of highly efficient technologies. Demand density variables are captured in the geospatial heterogeneity definition.
3. *Integration of socioeconomic factors*: Global socioeconomic, development, and demographic gridded datasets including GDP, GDPpc, HDI, and POP count offer a comprehensive understanding of the socioeconomic context of the energy transition. The integration of these factors allows for a more nuanced analysis of energy consumption patterns in relation to economic development and POP density. These variables are captured in the definition of energy consumers heterogeneity, diversity and evolution.
4. *Calibration and validation*: The calibration of socioeconomic datasets using additional geospatial datasets ensures that the models accurately represent real-world conditions. This calibration step is essential for validating the accuracy of the models and improving their reliability in predicting energy consumption patterns. This article presents three datasets to support validation of the energy consumers parametrisation.
5. *Climate impact assessment*: The inclusion of SH, WH, SC, and TE datasets, particularly at an hourly-seasonal resolution, enables a detailed assessment of the impact of climate variations on energy consumption. This information is crucial for understanding climate-related vulnerabilities and developing adaptive strategies.
6. *Economic modelling*: The integration of GDP, GDPpc, and other economic indicators allows for a robust economic modelling component. This enables policymakers and researchers to evaluate the economic implications of different energy consumption scenarios, helping in the formulation of sustainable and economically viable energy policies.
7. *Population dynamics*: POP count data at a 1 km² resolution provides insights into population distribution patterns. Understanding the spatial distribution of populations helps in predicting future energy demand trends, particularly in areas experiencing rapid population growth or urbanization.
8. *Scenario planning*: The combination of fine-grained energy demand datasets with socioeconomic and demographic information enables scenario planning. Stakeholders can assess the potential impact of different development scenarios, policy interventions, and climate change on energy consumption and make informed decisions.
9. *Resource allocation*: With detailed information on energy consumers, policymakers can allocate resources more efficiently, targeting investments and infrastructure development where they are needed the most.
10. *Consumer-led Policy design*: The insights gained from the integrated models can inform the design of energy, climate, and economic policies from the perspective of consumer realities. Policymakers can tailor interventions to address specific challenges in different regions, promoting sustainable development and energy efficiency for those that drive a just and equitable transition.

For users of these datasets, the parametrisation of energy consumers using geoAI and ABM foundations, along with the integration of diverse geospatial datasets, enhances the precision and applicability of energy, climate, and economic models. This approach allows for a comprehensive analysis of the complex interactions between energy consumption, socioeconomic factors, and environmental considerations, ultimately supporting informed decision-making for sustainable development.

## Supplementary information

Supplementary Information

## Data Availability

The algorithms and equations applied in this investigation have been previously disclosed. Three free and open-source platforms were employed in this research: (1) R Statistical Software and Programming Language, (2) Quantum GIS (QGIS) software, and (3) Python language. Various R Packages designed for geospatial big data analytics were employed in this study. QGIS served the purpose of data exploration due to its capabilities in viewing, editing, and analysing geospatial data. Python served as the development programmatic environment for the MUSE model available in Giarola, *et al*.^36^. The MUSE-RASA model^3^ was developed by integrating the R-based geospatial RASA model with the Python-based MUSE model. The R code use to generate the analysis of this article is provided in the following source^37^ [10.5281/zenodo.10594766].
