# Supplementary material for: Global datasets of geospatial-AI-resolved energy consumers including climate-driven energy demands, geographical and socioeconomic realities for a transition reset
Source: Sci Data. 2024 Dec 19;11:1408. doi: 10.1038/s41597-024-04277-x (PMC11659562; doi:10.1038/s41597-024-04277-x)
Supplement: Supplementary file 1 — Supplementary Information [file 41597_2024_4277_MOESM1_ESM.docx]

**Table S1:** **The list of countries by each region used in MUSE is reported here. The breakdown of regions used in the Energy Technology Perspectives of the IEA is considered.**

| USA | United States |
| --- | --- |
| CAN | Canada |
| MEX | Mexico |
| CHL | Chile |
| AUS | Australia, New Zealand |
| JPN | Japan |
| KOR | South Korea |
| ISR | Israel |
| Other Europe, OE2 | Switzerland, Turkey |
| ISL | Iceland |
| NOR | Norway |
| DNK | Denmark |
| FIN | Finland |
| SWE | Sweden |
| EU18 ( | Austria, Belgium, Czech Republic, France, Germany, Greece, Hungary, Ireland, Italy, Luxembourg, Netherlands, Poland, Portugal, Slovak Republic, Spain, United Kingdom, Slovenia, Estonia |
| EU7 | Bulgaria, Croatia, Cyprus, Latvia, Lithuania, Malta, Romania |
| Other European Transition Economies, OETE | Albania, Belarus, Bosnia and Herzegovina, FYR of Macedonia, Gibraltar, Montenegro, Republic of Kosovo, Republic of Moldova, Serbia, Ukraine |
| RUS | Russia |
| ATE | Azerbaijan, Kazakhstan, Turkmenistan, Uzbekistan, Armenia, Georgia, Kyrgyzstan, Tajikistan |
| CHN | People's Republic of China, Hong Kong |
| IND | India |
| ASEAN | Brunei Darussalam, Cambodia, Indonesia, Laos, Malaysia, Myanmar, Philippines, Singapore, Thailand, Viet Nam |
| Other Developing Asia, ODA | Afghanistan, Bangladesh, Bhutan, Chinese Taipei, Cook Islands, DPR of Korea, East Timor, Fiji, French Polynesia, Kiribati, Macau, Maldives, Mongolia, Nepal, New Caledonia, Pakistan, Papua New Guinea, Samoa, Solomon Islands, Sri Lanka, Tonga, Vanuatu |
| BRA | Brazil |
| OCSA  Latin America and Caribbean | Antigua and Barbuda, Argentina, Aruba, Bahamas, Barbados, Belize, Bermuda, Bolivia, British Virgin Islands, Cayman Islands, Colombia, Costa Rica, Cuba, Dominica, Dominican Republic, Ecuador, El Salvador, Falkland Islands, French Guyana, Grenada, Guadeloupe, Guatemala, Guyana, Haiti, Honduras, Jamaica, Martinique, Montserrat, Netherlands Antilles, Nicaragua, Panama, Paraguay, Peru, Saint Lucia, Saint Pierre et Miquelon, St. Kitts and Nevis, St. Vincent and the Grenadines, Suriname, Trinidad and Tobago, Turks and Caicos Islands, Uruguay, Venezuela |
| ZAF | South Africa |
| Other Africa, OAFR | All Africa except South Africa |
| MEA Middle East, MEA | Bahrain, Islamic Republic of Iran, Iraq, Jordan,  Kuwait, Lebanon, Oman, Qatar, Saudi Arabia, Syria, United Arab Emirates,  Yemen |
